# Application of Chaos Theory in the Assessment of Emotional Vulnerability and Emotion Dysregulation in Adults

**DOI:** 10.3390/brainsci10020089

**Published:** 2020-02-09

**Authors:** Cristina Ciuluvica (Neagu), Ioan Valeriu Grossu, Paolo Amerio

**Affiliations:** 1 Department of Psychological Sciences, Health, and Territory (DISPUTer), University G. D’Annunzio, Via dei Vestini, 66013 Chieti, Italy; 2 Faculty of Physics, University of Bucharest, P.O. Box MG 11, 077125 Bucharest-Măgurele, Romania; ioan.grossu@brahms.fizica.unibuc.ro; 3 Clinic of Dermatology, Department of Medicine and Aging Sciences, University G. D’Annunzio, Via dei Vestini, 66013 Chieti, Italy; p.amerio@unich.it

**Keywords:** Chaos theory, Lyapunov Function, Butterfly Effect, emotions, emotion regulation

## Abstract

In this work, we propose an interdisciplinary chaos analysis of emotion dysregulation (ED) and emotional vulnerability in adults. One of the main goals was the assessment of incongruences that occur in the evaluation of one’s own emotional dysregulation mechanisms in the presence of an extremely weak stimulus (Butterfly Effect). Thus, we considered a “flavor” of the Lyapunov Function method based on the assumption that the effort of answering to the test is itself a small perturbation. In this context, we calculated the “instability coefficient” Δ defined as the Euclidean distance between the pairs of vectors that include similar and reverted items of a test. The relationship between Δ, ED, and emotional characteristics as quality (positive/negative) and type (trait/state) was highlighted. We hypothesized that a higher level of Δ should be significantly related with a higher ED and with the type and the quality of emotions. The results suggest that Δ is significantly correlated with trait emotions (positively with negative emotions, and negatively with positive ones) and with ED. Moreover, Δ significantly predicts ED in adults. Thus, we consider that this approach is promising with respect to the evolution of emotional mechanisms across time. The presence of an initial instability to a weak perturbation might predict future abnormal emotional functioning, which could put at risk the mental or psychosomatic systems.

## 1. Introduction

A new interdisciplinary research perspective has developed recently concerning integration of theories from physics and mathematics in understanding the functioning of biological and psychological systems. Approaches using the laws of entropy or the laws of chaos have found a growing application in human sciences research. There is a growing interest in using an interdisciplinary approach in emotional research [1,2,3]. Emotions are cultural and psychobiological adaptation processes, extremely complex, and allow each individual to react flexibly and dynamically to both internal and external (environmental) contingencies [4]. In the present work, we tried to apply chaos theory to assess emotion dysregulation and emotional instability. 

Deterministic chaos refers to a class of deterministic systems in which seemingly random behavior is the result of dynamics described by nonlinear differential or recurrence equations (e.g., the Logistic Map). An important characteristic of such systems is the high dependence on initial conditions (Butterfly Effect), that is, a very small change in the initial state is associated with large differences in later states [5,6]. Thus, although deterministic, the evolution in time of such systems cannot be analytically predicted and cannot be even numerically approximated due to the finite resolution of any computational system. In recent years, chaos theory proved to be an important step in the complex transformation of health and the care models of nowadays.

Emotions could be defined as multifaceted, whole-body responses that involve coordinated changes in the domains of subjective experience, behavior, and peripheral physiology. Emotions arise when an individual attends to a situation and evaluates it as relevant [7,8]. This definition presupposes a chronological sequence of events involving, first, a real or imaginary situation; second, attention to and evaluation of the situation (appraisal) by the individual; and, third, an emotional response. 

Emotion regulation (ER) refers to attempts individuals make to influence which emotions they have, when they have them, and how these emotions are experienced and expressed [9]. The prototype of emotion regulation is a deliberate, effortful process that seeks to override people’s spontaneous emotional responses. There are forms of ER that correspond to this prototype by drawing upon the same psychological and neurobiological systems that are involved in the effortful control of action and attention [10,11,12]. Other forms of emotion regulation are relatively automatic and effortless. Automatic emotion regulation (AER) is characterized by goal-driven changes to one’s emotional experience without making a conscious decision to do so and without engaging in deliberate control [13,14]. 

In opposition with the definition of ER, we define emotion dysregulation as the inability to flexibly respond to and manage emotions [15]. In the literature, ED is conceptualized as a process that involves four dimensions: awareness and understanding of emotions, acceptance of emotions, ability to control impulsive behaviors and behave in accordance with desired goals when experiencing negative emotions, and ability to flexibly use situationally appropriate emotion regulation strategies to modulate emotional responses as desired in order to meet individual goals and situational demands [16].

Our goal was to estimate the sensitivity on initial conditions (Butterfly Effect) in analyzing emotional dysregulation and emotional vulnerability. We start from the assessment of the incongruences in one’s own emotional evaluation and hypothesized that higher values of Δ (the “instability coefficient” defined below) should correspond to higher values of ED and major negative emotions. There is a significant relationship between Δ and emotion dysregulation mechanisms. Δ should be significantly related with the type (trait/state) and quality (positive/negative) of emotions and could predict emotion dysregulation in adults. A prediction model for ED (emotion dysregulation) from Δ, negative affect of trait (NAT), and positive affect of trait (PAT) was also considered.

## 2. Method

### 2.1. Sample

The sample comprised 220 individuals selected from the general adult population who participated as volunteers in the study: 120 (64%) females, ranged 25 to 75 years of age, mean age 52.87, SD = 2.04. The study was conducted in Italy, Abruzzo region, between October 2017 and January 2019 in accordance with ethical standards on human experimentation and with the Helsinki Declaration of 1975, as revised in 1983. 

Inclusion criteria were age (over 18) and education (> 8 years). Exclusion criteria were inability to complete the assessment for different reasons (age, culture, education, and the presence of degenerative diseases such as dementia) and/or failure to give informed consent. 

All participants signed the informed consent form and completed a questionnaire regarding their demographic data. The demographic characteristics of the sample are presented in Table 1.

### 2.2. Instruments 

A research protocol consisting of three self-reported questionnaires (validated in Italian population) assessing emotion dysregulation mechanisms and the quality and type of emotions was applied [16,17,18,19].

Difficulties in Emotion Regulation Scale (DERS; Gratz & Roemer, 2004) [16,17] is a 36-item multidimensional self-report measure, subdivided into six subscales:

1. Nonacceptance of emotional responses (ACCEPTANCE);
2. Goals—difficulties engaging in goal-directed behavior when experiencing negative emotions (GOALS);
3. Impulse—impulse control difficulties when experiencing negative emotions (IMPULSE);
4. Awareness—lack of emotional awareness (AWARENESS);
5. Strategies—limited access to emotion regulation strategies that are perceived as effective (STRATEGIES);
6. Clarity—lack of emotional clarity (CLARITY), assessing the individual’s characteristic patterns of emotion dysregulation. Items are rated on a 5-point Likert-type scale (from 1 = almost never to 5 = almost always).

The total score on DERS indicates a global value of ED. A high total score indicates a high level of ED. High scores indicate the presence of a major difficulty in emotion regulation. In the current sample, internal consistency ranged between 0.74 for awareness and 0.82 for strategies and acceptance. 

Reverse-scored items were numbered 1, 2, 6, 7, 8, 10, 17, 20, 22, 24, and 34. The measure yielded a total score (SUM) as well as scores on six subscales. 

Positive and Negative Affect Schedule of Trait (PANAS–TRAIT) is a version of the Positive and Negative Affect Schedule (PANAS; Watson, Clark, and Tellegen, 1988) [18,19] that evaluates the emotions that one generally proves (trait tendency to experience positive or negative emotions). Results were recorded as positive affect of trait (PAT) and negative affect of trait (NAT). Cronbach’s α in the present sample ranged between 0.74 for PAT and 0.82 for NAT. 

Positive and Negative Affect Schedule of STATE (PANAS–STATE) is another version of the Positive and Negative Affect Schedule (PANAS; Watson, Clark, and Tellegen, 1988) [18,19] that evaluates the emotions that one proves in the assessment moment (positive or negative emotional reactivity in a specific situation). Results were recorded as positive affect of state (PAS) and negative affect of state (NAS) [20]. Cronbach’s α in the present sample ranged between 0.71 for PAS and 0.86 for NAS.

PANAS is a 20-item questionnaire: 10 items measure positive affect and 10 items measure negative affect. The positive affects are interested, excited, strong, enthusiastic, proud, alert, inspired, determinate, attentive, and active. The negative affects are distressed, upset, guilty, hostile, irritable, ashamed, nervous, jittery, and afraid. PANAS is one of the most widely used measures of affectivity and has been reported to have excellent psychometric proprieties with Italian samples. It was designed to measure affect in various contexts such as today, the past day, week, or year, or in general (on average). Thus, the scale can be used to measure state affect, dispositional or trait affect, emotional fluctuations throughout a specific period, or emotional responses to events. Each item is rated on a five-point Likert scale of 1 (not at all) to 5 (very much). High scores mean more frequent use of one kind of emotion. The measure has been used mainly as a research tool in group studies but can be utilized within clinical and nonclinical populations as well [18,19].

## 3. Procedure

The participants were asked to evaluate their difficulties in emotion regulation by answering the Difficulties in Emotion Regulation Scale (DERS) items. The evaluation was conducted in the presence of a clinical psychologist with experience in assessing emotional mechanisms. Before starting the self-evaluation, the participants were informed regarding the aim of the evaluation and the type of questionnaires. The order of the questionnaires was also specified, participants were asked to respect the order of the questionnaires in the protocol starting with DERS and continuing with PNAS–TRAIT, PANAS–STATE, and to the end to complete the demographic schedule. The evaluation protocol was built considering the semantic characteristics of items (accessible language and formulation) and the mean time involved to complete a protocol in order to not influence the attention process (15 min). 

We analyzed the incongruence that occurs in the evaluation of one’s own emotional dysregulation mechanisms in the presence an extremely weak stimulus (Butterfly Effect). We also considered that two core components of emotion regulation are strongly involved in the emotional evaluation process: emotional awareness (AWARENESS) and the access to emotion regulation strategies that are perceived as effective (STRATEGIES). In this context, the difficulties in maintaining a coherent emotional evaluation were investigated not only by scoring incoherence, but also by the direct assessment of incongruences in the evaluation of one’s own emotional mechanisms (reflected by Δ values). 

### 3.1. Lyapunov Function

Inspired by existing similar studies on both artificial and natural neural networks [20,21,22], for estimating the instability of perturbations (Butterfly Effect) we considered a “flavor” of the Lyapunov Function method, which supposes monitoring the evolution in time of two identical systems, separated by an infinitesimal dissimilarity in initial conditions. In the context of social sciences, one first difficulty comes from implementing an initial small perturbation. On the other hand, repeating questionnaires at different time intervals involves the presence of various, high-amplitude events in the life of each subject, which is incompatible with the proposed method. As an alternative solution, we started from the idea that the effort of answering to the test is itself a small perturbation in evaluating one’s own emotional mechanisms, resulting in different answers to equivalent questions. Thus, based on the Lyapunov Function method [20,23,24], we implemented the “instability coefficient”, defined as the Euclidean distance between two vectors comprising similar and reverse items:

(1) $$\Delta =\sqrt{\sum_{i}{\left(x_{i}-x_{ci}\right)}^{2}},$$

where *x_i_* is the item *i* score, and *x_ci_* is the score of the corresponding item (inverted for reverse items). 

For example, in the CLARITY cluster of DERS the (item, reverse item) pairs (components) are Item 1 and Item 9; and Item 4 and Item 7:

Item 1 (Time 1a) I am clear about my feelings. Item 9 (Time 2a) I am confused about how I feel;

Item 4 (Time 1b) I have no idea how I am feeling. Item 7 (Time 2b) I know exactly how I am feeling. 

Each similar item component also represents two different time points when the person self-evaluates her/his emotional mechanisms. In this context, the order of items should be carefully designed too. As the distance between all DERS similar/reverted items was reasonable, we considered rebalancing it was not necessary.

The distance at the component level reflects the degree to which the person did not maintain a coherent response when asked to self-evaluate the emotional mechanisms at two different points in time. By minimizing the unwanted effects (e.g., attention—train participants and conduct the evaluation in the presence of a clinical psychologist; cognitive issues—consider appropriate exclusion criteria for participants), we consider that the temporal distance between similar items (which includes the effort of answering to intermediary items) and the corresponding semantic differences (slightly different formulation or negation of the initial item) become the main disturbing stimuli with weak intensity of emotional awareness that could lead to important disturbances of self-evaluation. 

The correspondence between all similar items of DERS is presented in Table 2. Only 5 of 6 DERS clusters presented a correspondence between similar items: goals, impulse, awareness, strategies, and clarity. The nonacceptance cluster did not have corresponding items. Each of its 6 components investigated the presence of a specific emotion that could not be matched with any of the other 5: angry, embarrassed, ashamed, weak, guilty, and irritated.

The structure of the Difficulties in Emotion Regulation Scale (DERS) clusters and the general structure of DERS are also presented (Supplementary Tables S1 and S2).

### 3.2. Statistical Analysis

Descriptive analyses were run to determine means, standard deviations, and ranges. All the variables were checked for normality (Table 3). Δ, ED, PAT, NAT, PAS, and the DERS components (ACCEPTATION, GOALS, IMPULSE, AWARNESS, STRATEGIES, CLARITY) were normally distributed, while NAS was not normally distributed. To assess the significance of the relationship between the emotional instability coefficient values and ED, PAT, NAT, and PAS, Pearson correlation analysis was used. To assess if there was a significant relationship between NAS (not normally distributed) and the emotional instability coefficient values, Spearman correlation was used. For investigating the relation between emotional factors and Δ, and between Δ and ED, two stepwise regression analyses were performed in order to identify the better model that could explain the variance of the dependent variables. A simple linear regression was performed to assess the relation between Δ as unique independent variable and ED as predicted variable. All statistical analyses were provided by SPSS Statistics version 21 (IBM).

## 4. Results

As a primary analysis, the existence of a significant relationship between the instability coefficient Δ and both age and emotional aspects (dysregulation mechanisms, positive and negative affectivity of state and trait) was investigated. The results suggest that there is a significant, positive, linear relation between both Δ and ED (measured as total score of DERS) and Δ and the ED’s mechanisms, measured as the scores of the six subscales of DERS (ACCEPTATION, GOALS, IMPULSE, AWARNESS, STRATEGIES, CLARITY); Pearson’s r between 0.245 (for GOALS) and 0.457 (for STRATEGIES), *p* < 0.001. 

Age was not linearly associated with Δ. There were no significant linear relations between age and ED mechanisms (r = −0.011, *p* > 0.05). (Table 4)

The relation between gender and Δ was investigated using the analysis of variance between two independent groups (T test). The results suggest that there were no significant differences in Δ values between the two gender groups.

To investigate the significance of the relationship between Δ, and the positive and negative affectivity of state and trait, Pearson’s and Spearman’s correlation analyses were used. The results are presented in Table 2. The results verified both a significant, positive, linear correlation between Δ and NAT (0.250, *p* < 0.001) and a significant, negative, linear correlation between Δ and PAT (−0.191, *p* < 0.001). The results suggest that high values of Δ corresponded to high NAT values and low values of PAT. At the same time, higher instability was significantly correlated with low positive emotions (Table 5). The higher values of negative emotions (NAT, NAS) were significantly correlated with emotion dysregulation (ED) (Table 6). ED was also significantly negatively correlated with PAT. 

### Emotional Factors Associated with Instability Coefficient Δ

In order to better assess the emotional mechanisms associated with Δ, a stepwise linear regression was run. All significant dimensions (with respect to the bivariate analysis) of DERS and ED were included in the stepwise analysis, as predictors, to choose the best combination of dysregulation mechanisms that could predict Δ. The results suggest that only two variables significantly predicted Δ: limited access of emotion regulation strategies (STRATEGIES) and lack of emotional awareness (AWARENESS); F(2,198) = 28.972, *p* < 0.0001, *R*^2^ = 0.23. The model having STRATEGIES and AWARENESS as independent variables explained 23% of Δ variance (Table 7).

To investigate the relationship between ED, Δ, and associated emotional factors, another stepwise linear regression was run, having ED as the dependent variable and all variables significantly linearly related with ED as predictors: Δ, NAT, PAT, and NAS. The results suggest that the best combination of factors that could predict ED was represented by a model with three variables: NAT, Δ, and PAT. These variables statistically significantly predicted ED; F(3,217) = 36,589, *p* < 0.0001, *R*^2^ = 0.37, which indicated that 37% of ED variance was explained by the model. Inspection of regression coefficients indicated that all three variables (Δ, NAT, PAT) were significant predictors of ED (Table 8). 

The results of a simple linear regression analysis considering ED as a dependent variable and Δ as a unique independent variable suggested that Δ explained 19% of the ED variance; F(1,220) = 46,383, *p* < 0.0001, *R*^2^ = 0.19. 

## 5. Discussion

The present study proposes an interdisciplinary approach to investigate an individual’s instability to disturbance in evaluating their own emotional mechanisms when an extremely weak stimulus occurs. Starting from the existing chaos analyses on Artificial Neural Networks, where the complexity comes from interconnecting multiple nonlinear computational units (neurons), we considered finding similar behaviors in humans as a plausible assumption. Emotion regulation is only one of the psychological subjects with a growing interest in such an interdisciplinary approach. An important characteristic of nonlinear systems is the high dependence on initial conditions (Butterfly Effect) and could be estimated using the Lyapunov Exponent method, which is based on monitoring the evolution in time of the distance between two identical systems with a small difference (perturbation) in initial state. Although fine for numerical simulations, this method encounters significant difficulties in the field of social sciences. In this context, we tried to find an alternative solution based on the idea that the effort of answering a questionnaire represents, itself, a perturbation. Thus, one could estimate the instability in answering by measuring the Euclidean distance between the vectors that include the similar and reverted items of a test. 

In order to investigate the emotion dysregulation mechanisms, we applied three specific questionnaires (DERS, PANAS TRAIT, and PANAS STATE) to 220 subjects. To answer the DERS items, the subjects evaluated their own emotional mechanisms in a past period. The emotional evaluation process involves conscious (deliberate) mechanisms directly accessible through the cognitive processes of scoring the DERS items. Cognitive evaluation is an important process involved in both emotion generation and emotion regulation processes and is highly related with other emotion regulation mechanisms as emotional awareness. In this context, we measured the Euclidian distance between similar DERS items and defined the “instability coefficient” Δ. According to the method that we used in the Δ measure (Lyapunov Exponent method), a fast growth (e.g., exponential) is associated with a high instability of the system on perturbations. As in our context the investigated mechanisms are emotional mechanisms, we suggest that Δ could be related to emotional instability, understood not as daily symptomatology in the common definitions, but as the potential to have an abnormal emotional functioning in time and high vulnerability to emotional stimuli, which could invalidate the system’s functioning. 

The Butterfly Effect represents the sensitive dependence on initial conditions, that is, a very small change in one state in a deterministic nonlinear system is associated with large differences in a later state. Starting from the Butterfly Effect conceptualization, and considering the human body as a whole system including different subsystems in interaction, we suggest that Δ could be measured to investigate psychosomatic functioning—a small change in the emotional system could generate in time an important reaction in the somatic system. Future research could be provided to assess Δ in different clinical samples.

As recent literature highlights, most contemporary dual-process models contrast automatic (also called nonconscious, implicit, or impulsive) processes with deliberate (also called controlled, conscious, explicit, or reflective) processes. Deliberate processing requires attention resources, is volitional, and is driven by explicit goals. In contrast, automatic processing is initiated by the simple registration of sensory inputs, which in turn activates knowledge structures (schemas, scripts, or concepts) that then shape other psychological functions [25,26]. In our research, since the intensity of the stimuli that we considered in the Δ measure was weak and was not perceived as important disruptors requiring special attention and concentration adaptation to respond to the above-mentioned changes, we believe that the mechanisms that interfere with disruption are automatic (unconscious).

However, it is important to notice that this measure can be connected to multiple aspects that cannot be completely excluded. Thus, for minimizing attention effects, we chose only the subjects that understood and accepted the importance of the evaluation and the tests that were monitored.

The emotional factors significantly related with Δ were assessed using statistical analysis. The results suggest that Δ is significantly related with emotion dysregulation. Higher values of Δ resulted positively, correlated with both ED and emotion dysregulation components (ACCEPTATION, GOALS, IMPULSE, AWARNESS, STRATEGIES, CLARITY). At the same time, high values of Δ correspond to high NAT values and low values of PAT. This means that, in adult people, the tendency to have more frequent negative and less positive emotional responses is higher when the Δ is higher. Only two emotion dysregulation components resulted in Δ predictors: lack of emotional awareness (AWARENESS) and limited access to emotion regulation strategies perceived as efficient (STRATEGIES). These two emotion dysregulation mechanisms predicted 23% of Δ variance. The results suggest also an important relation between Δ and ED. Δ predicted 29% from ED variance as a single predictor and 38% in a model of multiple linear regression with NAT and PAT. 

These results encourage our hope that the described analysis could contribute to the prediction of future abnormal emotional reaction in adults that could put at risk their mental and psychosomatic systems functioning. This type of analysis of emotion regulation could be important in the assessment and understanding of emotional mechanisms in several pathological states [27,28]. The instability coefficient could be also used as a general evaluation validity check. Thus, high values of Δ are a warning indicator of potential issues to be further investigated by the evaluator.

## 6. Conclusions

The results of the present study suggest that the described model is promising regarding the evolution of emotional mechanisms across time. The presence of an initial instability to a weak perturbation might predict future abnormal emotional functioning, which could put at risk the mental or psychosomatic systems. Early identification of the probability of an abnormal functioning over time may find future applications in health and medical sciences, contributing to identify emotional risk and guide preventive interventions. 

## Figures and Tables

**Table 1:** Demographic Characteristics of the sample.

	*N*	Age (Years)	Gender	Working Status	Civil Status	Education (Years)
		Mean ± SD	Female *n* (%)	Workinge *n* (%)	Marriede *n* (%)	Mean ± SD
Total sample	220	52.87 ± 2.04	120 (64.21)	184 (86.34)	168 (69.43)	11.83 ± 3.92

**Table 2:** Item correspondence; R = reverse scoring.

Nr	Timee 1	Item Content	Nr	Timee 2	Corresponding Item Content
		**CLARITY**			
I1.R	1a	I am clear about my feelings.	I9	2a	I am confused about how I feel.
I4	1b	I have no idea how I am feeling.	I7.R	2b	I know exactly how I am feeling
		**GOALS**			
I13	1c	When I’m upset, I have difficulty getting work done.	I20.R	2c	When I’m upset, I can still get things done.
I18	1d	When I’m upset, I have difficulty focusing on other things	I26	2d	When I’m upset, I have difficulty concentrating.
		**IMPULSE**			
I14	1e	When I’m upset, I become out of control.	I32	2e	When I’m upset, I lose control over my behavior.
I3	1f	I experience my emotions as overwhelming and out of control.	I19	2f	When I’m upset, I feel out of control.
I24.R	1g	When I’m upset, I feel like I can remain in control of my behaviors.	I27	2g	When I’m upset, I have difficulty controlling my behaviors
		**AWARENESS**			
I2.R	1h	I pay attention to how I feel.	I6.R	2h	I am attentive to my feelings.
I8.R	1i	I care about what I am feeling	I34.R	2i	When I’m upset, I take time to figure out what I’m really feeling.
I8.R	1j	When I’m upset, I acknowledge my emotions	I10.R	2j	When I’m upset, I believe that my feelings are valid and important.
		**STRATEGIES**			
I15	1k	When I’m upset, I believe that I will remain that way for a long time.	I35	2k	When I’m upset, it takes me a long time to feel better.
I16	1l	When I’m upset, I believe that I will end up feeling very depressed.	I30	2l	When I’m upset, I start to feel very bad about myself.
I22	1m	When I’m upset, I know that I can find a way to eventually feel better	I28	2m	When I’m upset, I believe there is nothing I can do to make myself feel better.
I31	1n	When I’m upset, I believe that wallowing in it is all I can do.	I36	2n	When I’m upset, my emotions feel overwhelming.

**Table 3:** Minimum values, maximum values, and the skewness and kurtosis of normality coefficients.

Variable	Min	Max	Skewness	Std. Error	Kurtosis	Std. Error
Δ	0.000	11.090	0.255	0.171	0.687	0.340
ED	34	143	0.845	0.164	0.417	0.327
PAT	11	50	−0.349	0.165	0.176	0.328
NAT	10	47	1.046	0.165	0.723	0.328
PAS	11	50	−0.035	0.165	−0.089	0.329
NAS	10	41	1.740	0.165	2.973	0.329
ACCEPTATION	6	30	1.144	0.164	0.691	0.327
GOALS	5	25	0.467	0.164	−0.204	0.327
IMPULSE	8	30	1.571	0.164	1.908	0.327
AWARENESS	6	17	0.549	0.164	−0.506	0.327
STRATEGIES	7	34	0.538	0.164	−0.045	0.327
CLARITY	5	22	0.698	0.164	0.138	0.327
AGE	25	75	−0.192	0.163	−0.538	0.324

**Table 4:** Pearson correlations of Δ/DERS/AGE.

	Mean	SD	Δ	ACC	GOAL	STRAT	IMP	CLAR	AWAR	ED
Δ	4.801	1.840	-							
ACCEPTATION	12.27	5.825	0.285 **	-						
GOALS	12.11	4.548	0.245 **	0.619 **	-					
STRATEGIES	17.17	5.362	0.457 **	0.514 **	0.594 **	-				
IMPULSE	11.15	4.697	0.292 **	0.534 **	0.603 **	0.552 **	-			
CLARITY	10.23	3.853	0.321 **	0.373 **	0.311 **	0.414 **	0.323 **	-		
AWARENESS	7.25	3.071	0.254 **	0.117	0.012	0.220 **	−0.013	0.384 **	-	
ED	70.20	19.680	0.437 **	0.800 **	0.784 **	0.822 **	0.749 **	0.647 **	0.327 **	-
AGE	52.87	14.264	−0.011	0.060	0.033	0.047 **	−0.048	0.020	−0.021	0.029

** Correlation is significant at the 0.01 level (2-tailed).

**Table 5:** Pearson correlations of Δ/PANAS Trait and Δ/PANAS State.

	Mean	SD	Δ	PAT	NAT	PAS	NAS
Δ	4.801	1.840	-				
PAT	32.01	7.511	−0.191 **	-			
NAT	19.05	7.799	0.250 **	0.172 *	-		
PAS	29.23	7.723	−0.167 *	0.720 **	0.140 *	-	
NAS	15.26	6.445	0.057	0.006	0.567 **	0.193 **	-

** Correlation is significant at the 0.01 level (2-tailed). * Correlation is significant at the 0.05 level (2-tailed). PAT—tendency to positive emotions, NAT—tendency to negative emotions, PAS—positive emotion reactivity, NAS—negative emotion reactivity, Δ—the Euclidean distance in answering DERS items (instability coefficient).

**Table 6:** Pearson correlations of ED/PANAS Trait and ED/PANAS State.

	Mean	SD	ED	PAT	NAT	PAS	NAS
ED	72.20	19.20	-				
PAT	32.01	7.51	−0.207 **	-			
NAT	19.05	7.79	0.477 **	0.172 *	-		
PAS	29.23	7.72	−0.091	0.720 **	0.140 *	-	
NAS	15.26	6.44	0.247 **	0.006	0.567 **	0.193 **	-

**. Correlation is significant at the 0.01 level (2-tailed). *. Correlation is significant at the 0.05 level (2-tailed). ED—total value of emotion dysregulation (ED) measured as total score of DERS, PAT—tendency to positive emotions, NAT—tendency to negative emotions, PAS—positive emotion reactivity, NAS—negative emotion reactivity.

**Table 7:** Standard multiple regression analysis summary for variables predicting the instability coefficient (Δ).

Variables	B	SEB	β
STRATEGIES	0.132	0.021	0.416 ***
AWARENESS	0.082	0.037	−0.151 *

*R*^2^ = 0.23 (*N* = 220, *p* < 0.0001). * *P* < 0.05; *** *P* < 0.0001.

**Table 8:** Standard regression analysis summary for variables predicting emotion dysregulation. (ED).

Variables	B	SEB	β
Δ	2.897	0.713	0.253 ***
NAT	1.072	0.154	0.436 ***
PAT	−0.658	0.157	−0.252 ***

*R*^2^ = 0.37 (*N* = 220, *p* < 0.0001); *** *p* < 0.0001.

## Supplementary Materials

The following are available online at https://www.mdpi.com/2076-3425/10/2/89/s1, Table S1: DERS Clusters Structure; R = REVERSE SCORING, Table S2: Difficulties in Emotion Regulation Scale (DERS), Gratz & Roemer (2004); D = DIRECT SCORING (from 1 – almost never to 5 – almost always), R = REVERSE SCORING (from 5 – almost never to 1 – almost always).
