# Supplementary material for: Application of Chaos Theory in the Assessment of Emotional Vulnerability and Emotion Dysregulation in Adults
Source: Brain Sci. 2020 Feb 9;10(2):89. doi: 10.3390/brainsci10020089 (PMC7071830; doi:10.3390/brainsci10020089)
Supplement: Supplementary file 1 [file brainsci-10-00089-s001.pdf]

Supplementary Material: Application of Chaos Theory in the Assessment of Emotional Vulnerability and Emotion Dysregulation in Adults

Table S1. DERS Clusters Structure; R = REVERSE SCORING.

Scoring	Nr	Clusters structure
		**NONACCEPTANCE**
	I11.	When I'm upset, I become angry with myself for feeling that way.
	I12.	When I'm upset, I become embarrassed for feeling that way.
	I21.	When I'm upset, I feel ashamed at myself for feeling that way.
	I23.	When I'm upset, I feel like I am weak.
	I25.	When I'm upset, I feel guilty for feeling that way.
	I29.	When I'm upset, I become irritated at myself for feeling that way.
		**GOALS**
	I13.	When I'm upset, I have difficulty getting work done.
	I18.	When I'm upset, I have difficulty focusing on other things
R	I20.	When I'm upset, I can still get things done.
	I26.	When I'm upset, I have difficulty concentrating.
	I33.	When I'm upset, I have difficulty thinking about anything else.
		**IMPULSE**
	I3.	I experience my emotions as overwhelming and out of control.
	I14.	When I'm upset, I become out of control.
	I19.	When I'm upset, I feel out of control.
R	I24.	When I'm upset, I feel like I can remain in control of my behaviors.
	I27.	When I'm upset, I have difficulty controlling my behaviors
	I32.	When I'm upset, I lose control over my behavior.
		**AWARENESS**
R	I2.	I pay attention to how I feel.
R	I6.	I am attentive to my feelings.
R	I8.	I care about what I am feeling
R	I10.	When I'm upset, I acknowledge my emotions
R	I17.	When I'm upset, I believe that my feelings are valid and important.
R	I34	When I'm upset I take time to figure out what I'm really feeling.
		**STRATEGIES**
	I15.	When I'm upset, I believe that I will remain that way for a long time.
	I16.	When I'm upset, I believe that I will end up feeling very depressed.
R	I22.	When I'm upset, I know that I can find a way to eventually feel better
	I28.	When I'm upset, I believe there is nothing I can do to make myself feel better.
	I30.	When I'm upset, I start to feel very bad about myself.
	I31.	When I'm upset, I believe that wallowing in it is all I can do.
	I35.	When I'm upset, it takes me a long time to feel better.
	I36.	When I'm upset, my emotions feel overwhelming.
		**CLARITY**
R	I1.	I am clear about my feelings.
	I4.	I have no idea how I am feeling.
	I5.	I have difficulty making sense out of my feelings.

R	I7.	I know exactly how I am feeling
	I9.	I am confused about how I feel.

Table S2. Difficulties in Emotion Regulation Scale (DERS), Gratz & Roemer (2004); D = DIRECT SCORING (from 1 – almost never to 5 – almost always), R = REVERSE SCORING (from 5 – almost never to 1 – almost always).

Nr.	Scoring	Item content
I1	R	I am clear about my feelings.
I2	R	I pay attention to how I feel.
I3	D	I experience my emotions as overwhelming and out of control.
I4	D	I have no idea how I am feeling.
I5	D	I have difficulty making sense out of my feelings.
I6	R	I am attentive to my feelings.
I7	R	I know exactly how I am feeling.
I8	R	I care about what I am feeling.
I9	D	I am confused about how I feel.
I10	D	When I'm upset, I acknowledge my emotions.
I11	D	When I'm upset, I become angry with myself for feeling that way.
I12	D	When I'm upset, I become embarrassed for feeling that way
I13	D	When I'm upset, I have difficulty getting work done
I14	D	When I'm upset, I become out of control.
I15	D	When I'm upset, I believe that I will remain that way for a long time.
I16	D	When I'm upset, I believe that I will end up feeling very depressed.
I17	R	When I'm upset, I believe that my feelings are valid and important.
I18	D	When I'm upset, I have difficulty focusing on other things.
I19	D	When I'm upset, I feel out of control.
I20	R	When I'm upset, I can still get things done.
I21	D	When I'm upset, I feel ashamed at myself for feeling that way.
I22	R	When I'm upset, I know that I can find a way to eventually feel better.
I23	D	When I'm upset, I feel like I am weak.
I24	R	When I'm upset, I feel like I can remain in control of my behaviors
I25	D	When I'm upset, I feel guilty for feeling that way.
I26	D	When I'm upset, I have difficulty concentrating.
I27	D	When I'm upset, I have difficulty controlling my behaviors.
I28	D	When I'm upset, I believe there is nothing I can do to make myself feel better.
I29	D	When I'm upset, I become irritated at myself for feeling that way.
I30	D	When I'm upset, I start to feel very bad about myself.
I31	D	When I'm upset, I believe that wallowing in it is all I can do.
I32	D	When I'm upset, I lose control over my behavior.
I33	D	When I'm upset, I have difficulty thinking about anything else.
I34	R	When I'm upset I take time to figure out what I'm really feeling.
I35	D	When I'm upset, it takes me a long time to feel better.
I36	D	When I'm upset, my emotions feel overwhelming.
